# Supplementary figures and images for: Exploring chilling stress and recovery dynamics in C4 perennial grass of Miscanthus sinensis
Source: PLoS One. 2025 Jan 3;20(1):e0308162. doi: 10.1371/journal.pone.0308162 (PMC11698526; doi:10.1371/journal.pone.0308162)

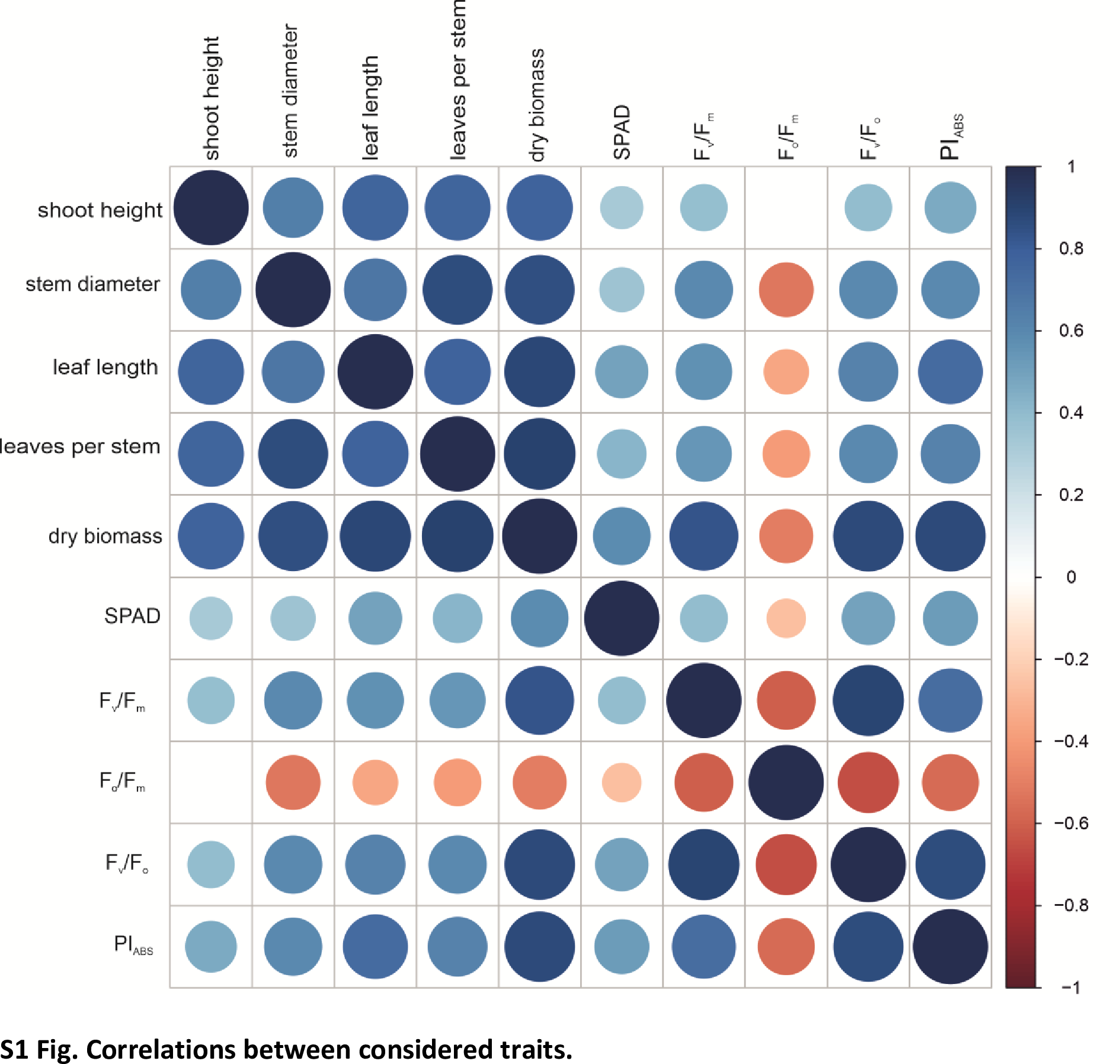

Supplement: S1 Fig — (TIF) [file pone.0308162.s001.tif]

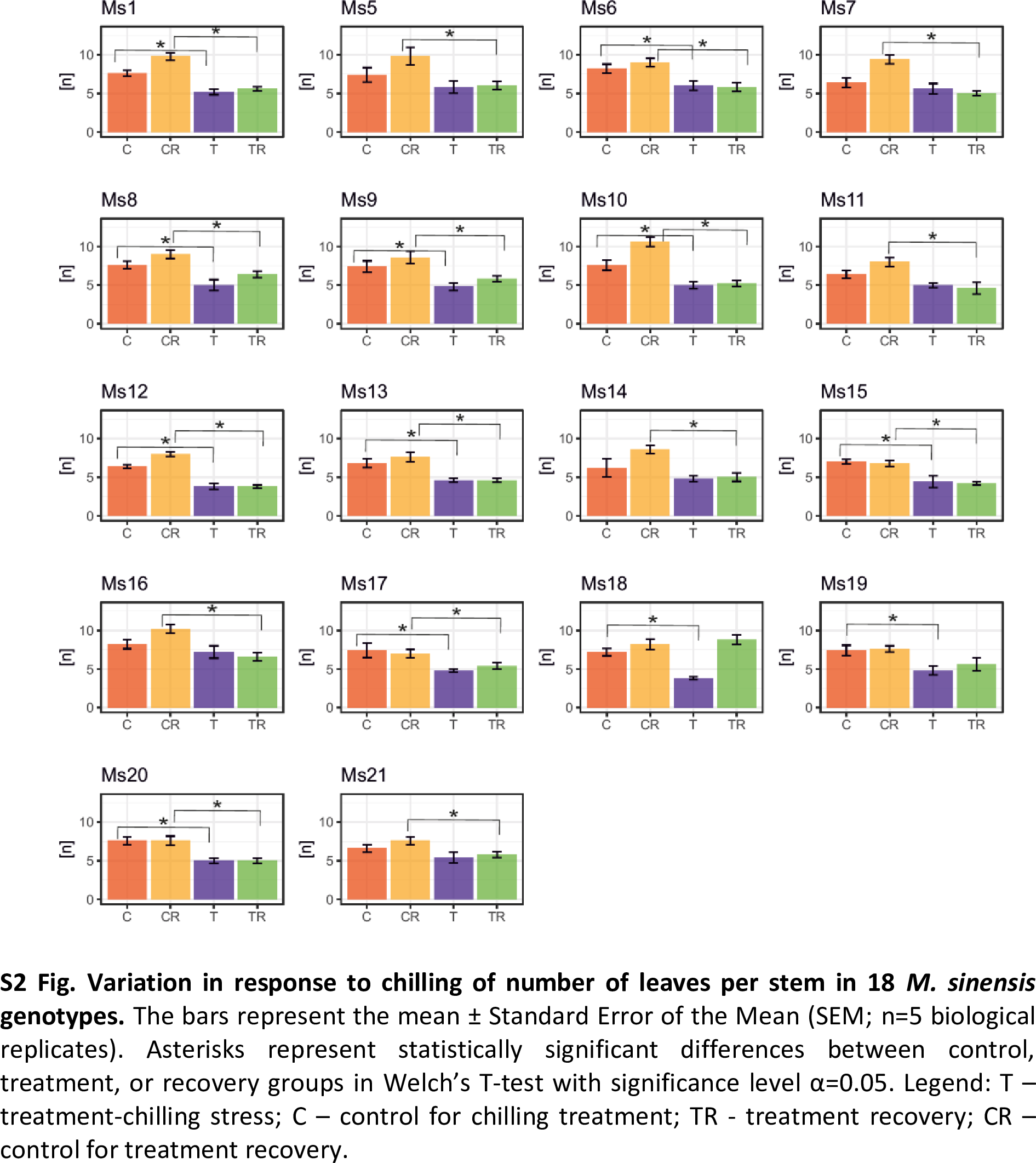

Supplement: S2 Fig — The bars represent the mean ± Standard Error of the Mean (SEM; n = 5 biological replicates). Asterisks represent statistically significant differences between control, treatment, or recovery groups in Welch’s T-test with significance level α = 0.05. T–treatment-chilling stress; C–control for chilling treatment; TR—treatment recovery; CR–control for treatment recovery. (TIF) [file pone.0308162.s002.tif]

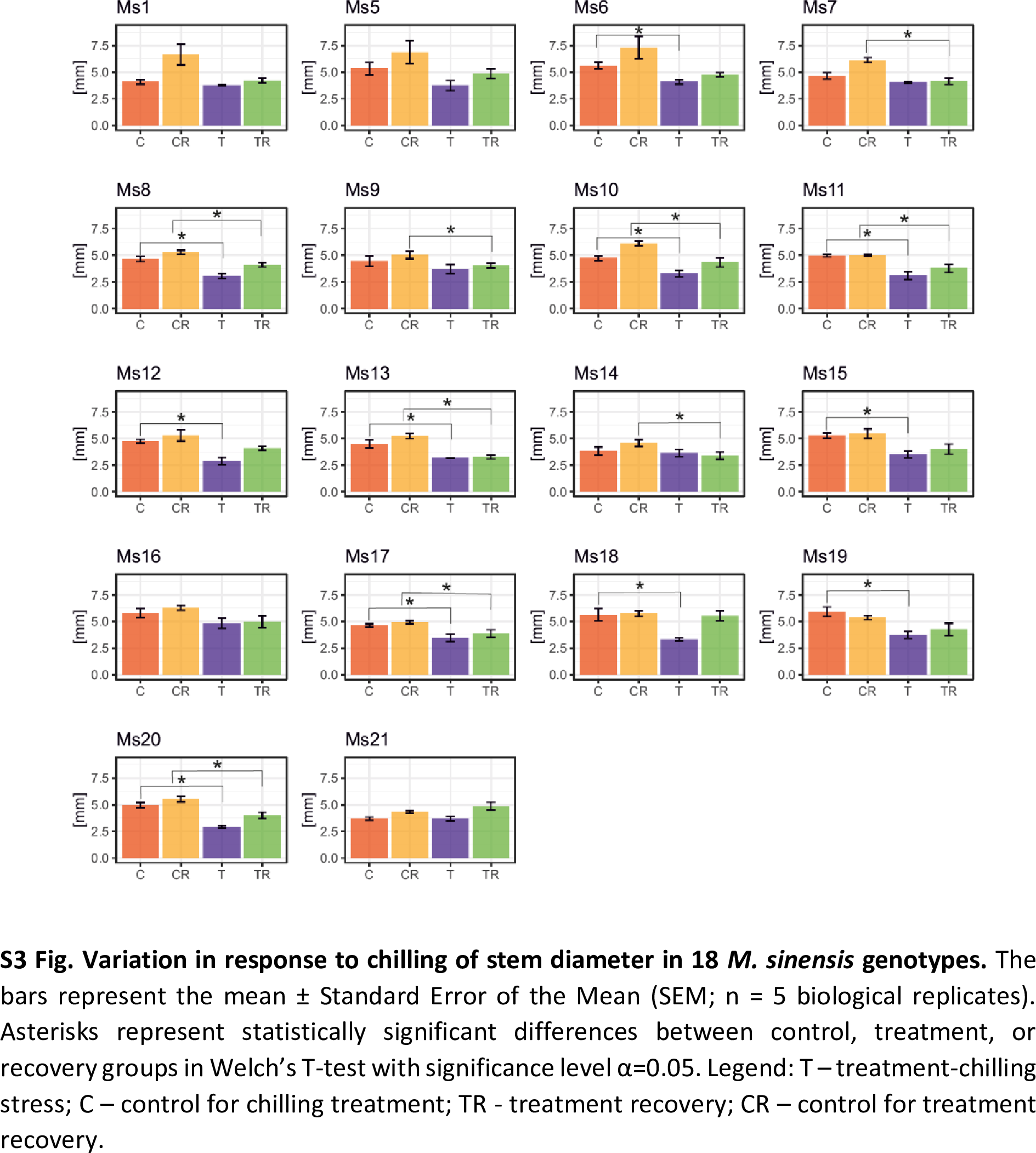

Supplement: S3 Fig — The bars represent the mean ± Standard Error of the Mean (SEM; n = 5 biological replicates). Asterisks represent statistically significant differences between control, treatment, or recovery groups in Welch’s T-test with significance level α = 0.05. T–treatment-chilling stress; C–control for chilling treatment; TR—treatment recovery; CR–control for treatment recovery. (TIF) [file pone.0308162.s003.tif]

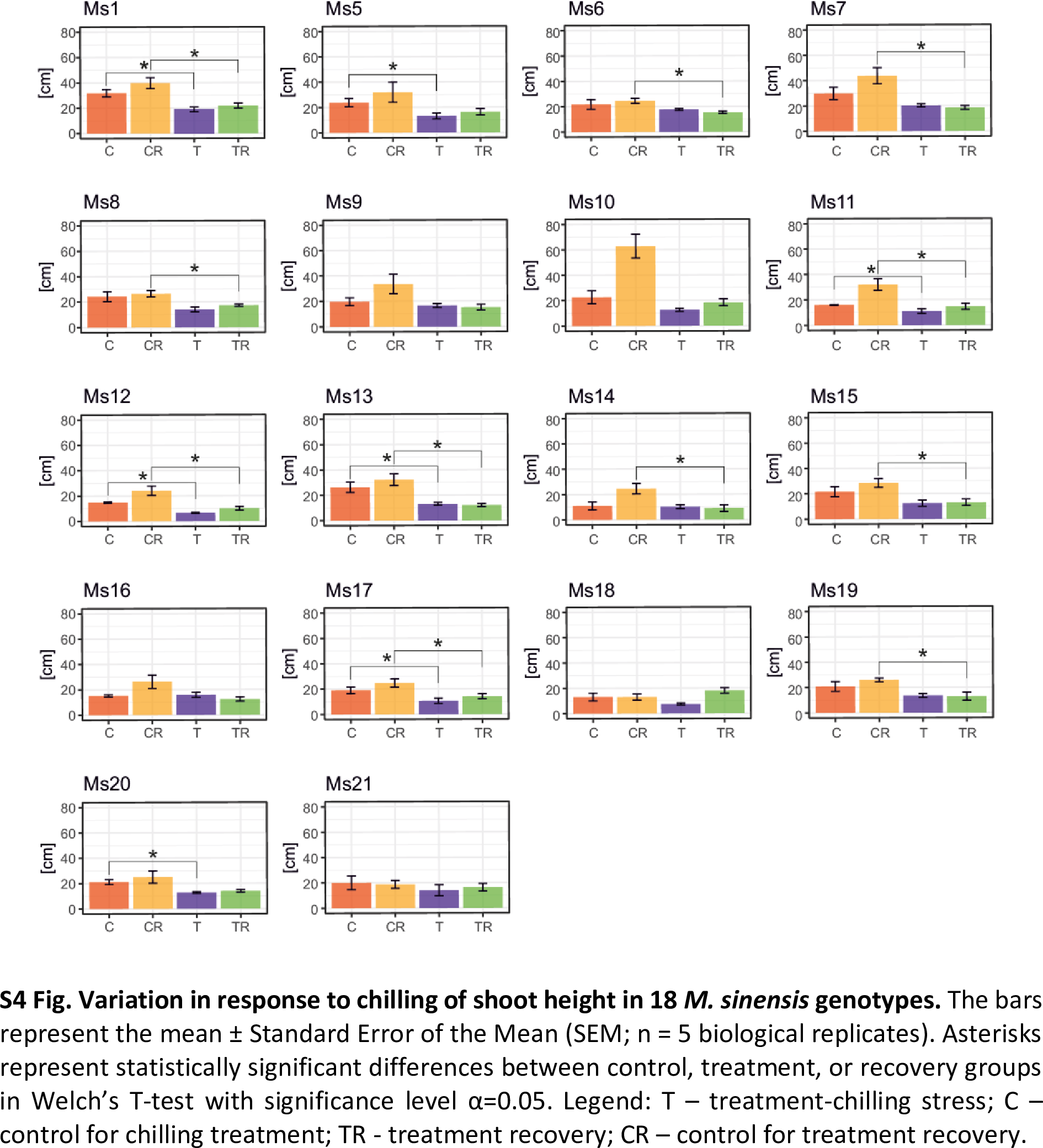

Supplement: S4 Fig — The bars represent the mean ± Standard Error of the Mean (SEM; n = 5 biological replicates). Asterisks represent statistically significant differences between control, treatment, or recovery groups in Welch’s T-test with significance level α = 0.05. T–treatment-chilling stress; C–control for chilling treatment; TR—treatment recovery; CR–control for treatment recovery. (TIF) [file pone.0308162.s004.tif]

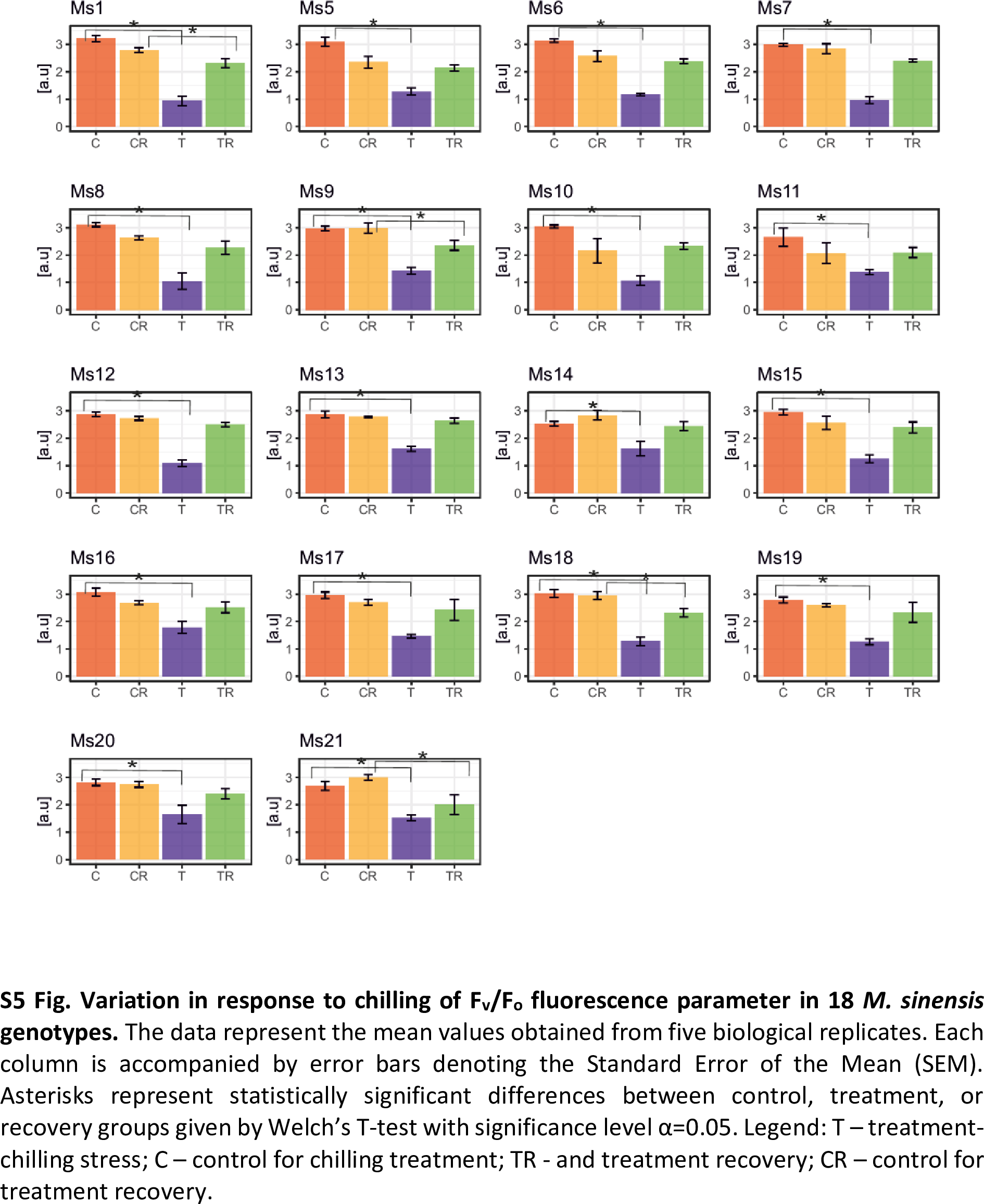

Supplement: S5 Fig — The data represent the mean values obtained from five biological replicates. Each column is accompanied by error bars denoting the Standard Error of the Mean (SEM). Asterisks represent statistically significant differences between control, treatment, or recovery groups given by Welch’s T-test with significance level α = 0.05. T–treatment-chilling stress; C–control for chilling treatment; TR—and treatment recovery; CR–control for treatment recovery. (TIF) [file pone.0308162.s005.tif]

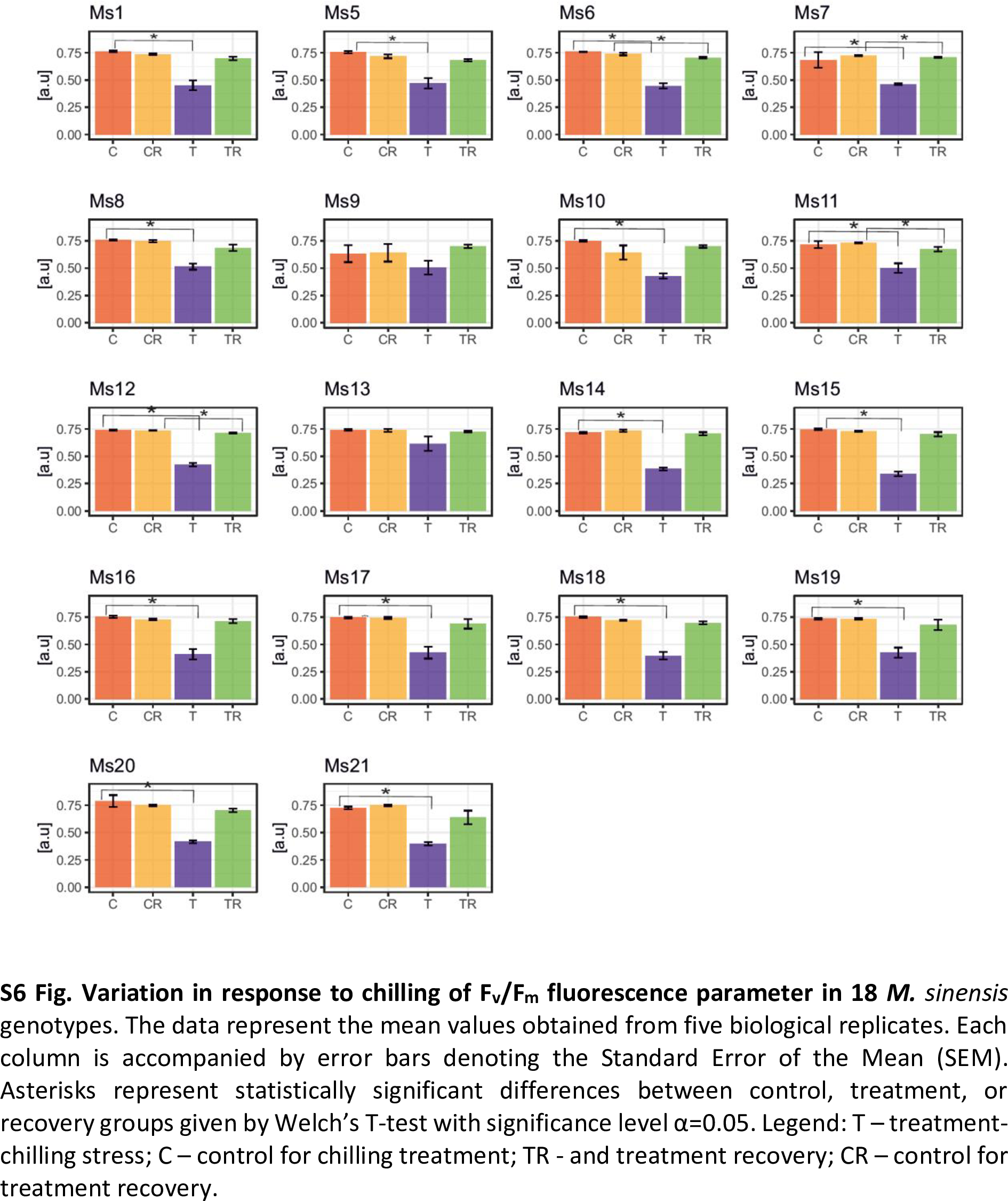

Supplement: S6 Fig — The data represent the mean values obtained from five biological replicates. Each column is accompanied by error bars denoting the Standard Error of the Mean (SEM). Asterisks represent statistically significant differences between control, treatment, or recovery groups given by Welch’s T-test with significance level α = 0.05. T–treatment-chilling stress; C–control for chilling treatment; TR—and treatment recovery; CR–control for treatment recovery. (TIF) [file pone.0308162.s006.tif]

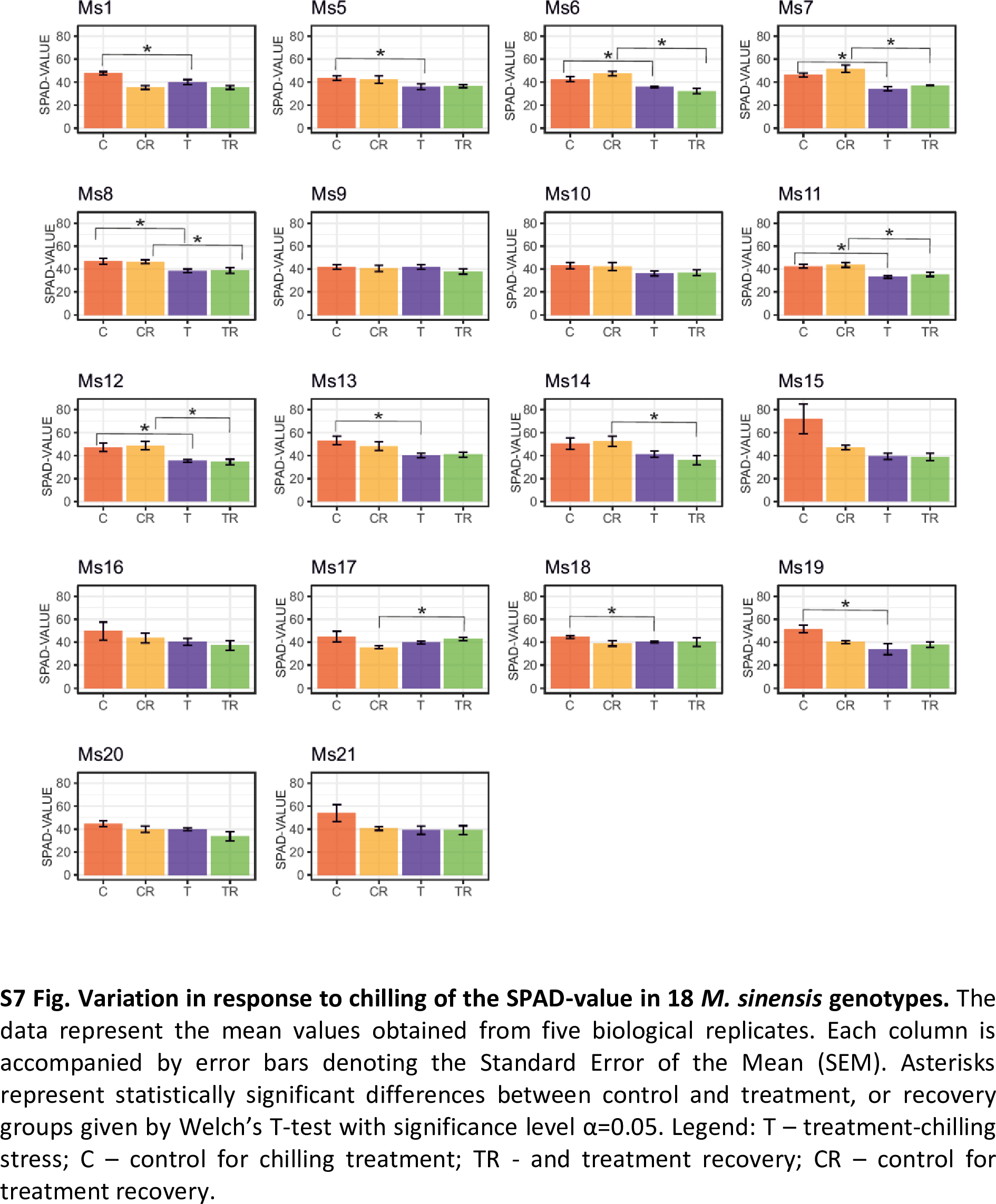

Supplement: S7 Fig — The data represent the mean values obtained from five biological replicates. Each column is accompanied by error bars denoting the Standard Error of the Mean (SEM). Asterisks represent statistically significant differences between control and treatment, or recovery groups given by Welch’s T-test with significance level α = 0.05. T–treatment-chilling stress; C–control for chilling treatment; TR—and treatment recovery; CR–control for treatment recovery. (TIF) [file pone.0308162.s007.tif]

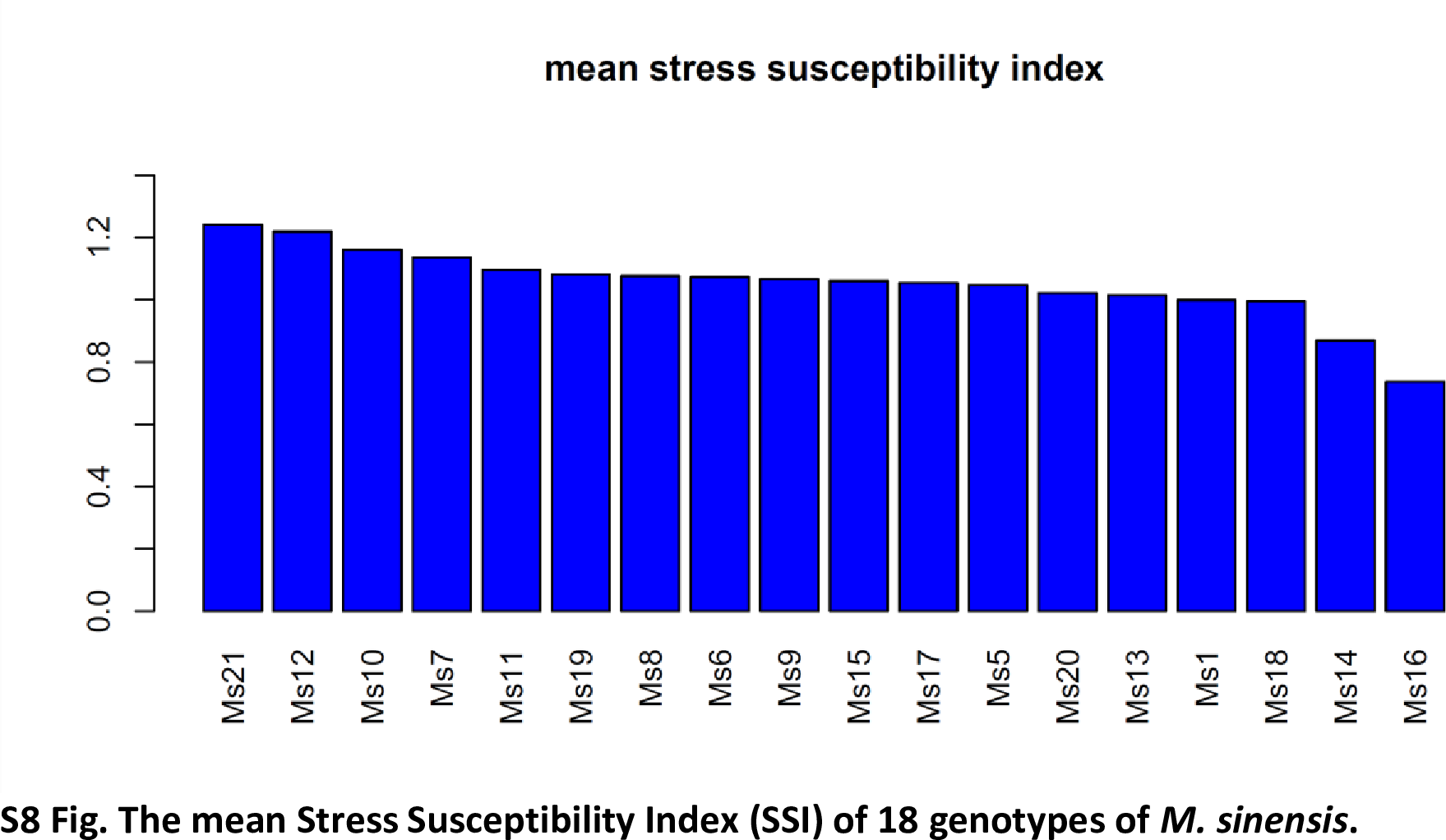

Supplement: S8 Fig — (TIF) [file pone.0308162.s008.tif]

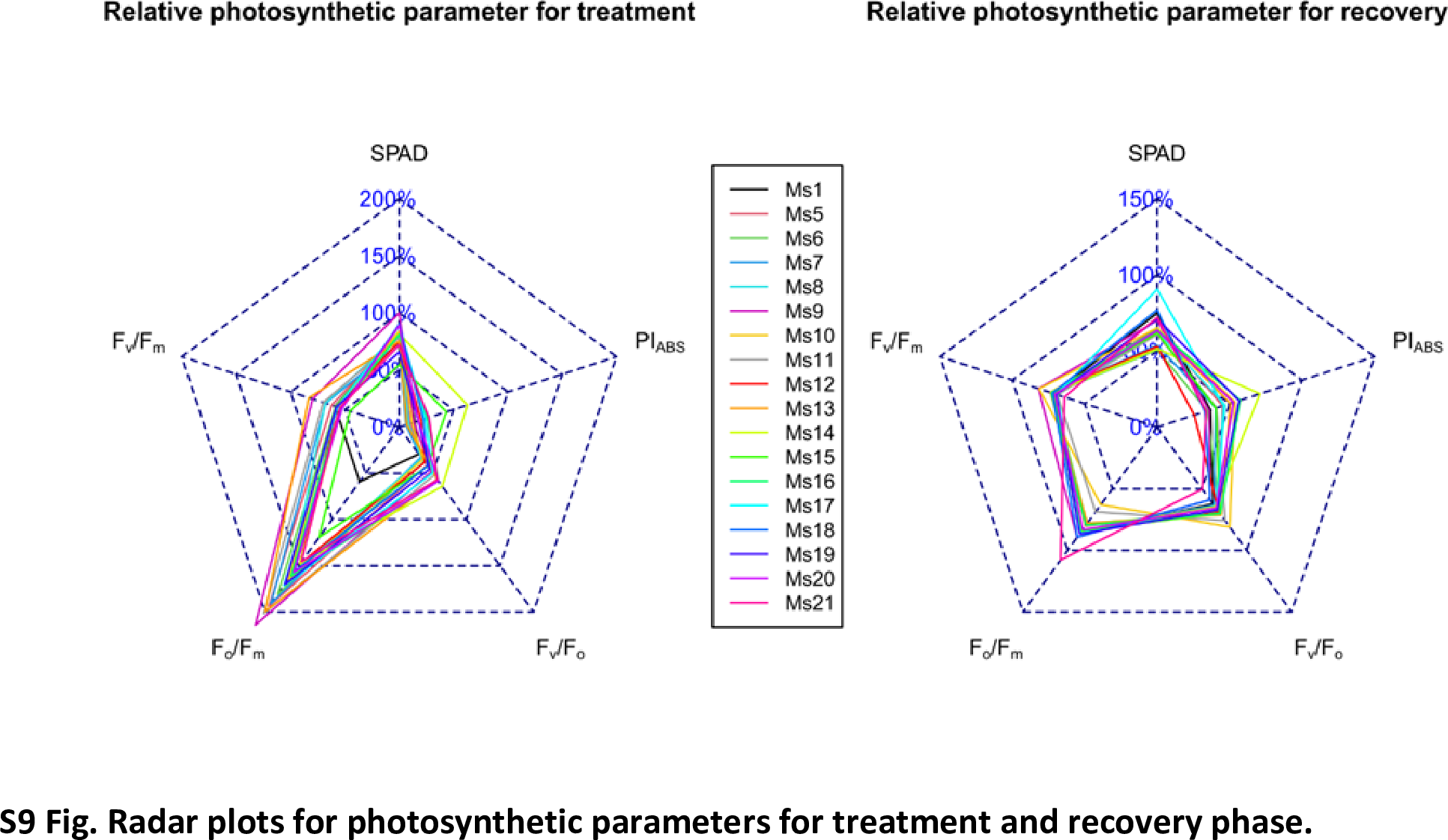

Supplement: S9 Fig — (TIF) [file pone.0308162.s009.tif]
