# Supplementary material for: Exploring chilling stress and recovery dynamics in C4 perennial grass of Miscanthus sinensis
Source: PLoS One. 2025 Jan 3;20(1):e0308162. doi: 10.1371/journal.pone.0308162 (PMC11698526; doi:10.1371/journal.pone.0308162)
Supplement: Attachment — Submitted filename: Response to Reviewers.docx [file pone.0308162.s011.docx]

To Editor,

Thank you for considering the manuscript (Ms. Ref. No.: PONE-D-24-12204), titled “Exploring Chilling Stress and Recovery Dynamics in C4 perennial grass of Miscanthus sinensis” for publication in POLS ONE. We extend our gratitude to the anonymous reviewers for their insightful comments and suggestions, which significantly enhanced the manuscript. All the comments that reviewers brought are addressed in the revised version of our manuscript. At the request of reviewers, in the revised version of the manuscript two new figures have been added and the text has been trimmed to make it more focused.

Journal Requirements and both Reviewers' comments in black. Our responses in blue.

Journal Requirements:

When submitting your revision, we need you to address these additional requirements.

1. Please ensure that your manuscript meets PLOS ONE's style requirements, including those for file naming. The PLOS ONE style templates can be found at 
https://journals.plos.org/plosone/s/file?id=wjVg/PLOSOne_formatting_sample_main_body.pdf and 
https://journals.plos.org/plosone/s/file?id=ba62/PLOSOne_formatting_sample_title_authors_affiliations.pdf

A: The text formatting has been changed in the revised manuscript to meet PLOS ONE style requirements.

2.  We note that the grant information you provided in the ‘Funding Information’ and ‘Financial Disclosure’ sections do not match.

When you resubmit, please ensure that you provide the correct grant numbers for the awards you received for your study in the ‘Funding Information’ section.

A: We apologize for this inconsistency which has been corrected in the revised version of the manuscript.

The grant number was checked.

3. Thank you for stating the following financial disclosure: 
"This work was supported by the National Center Science Poland (NCN) [grant 218/29/N/NZ9/00854]. "

Please state what role the funders took in the study.  If the funders had no role, please state: "The funders had no role in study design, data collection and analysis, decision to publish, or preparation of the manuscript." 
If this statement is not correct you must amend it as needed. 
Please include this amended Role of Funder statement in your cover letter; we will change the online submission form on your behalf.

A: Thank you for this comment. The statement has been added.

This work was supported by the National Center Science Poland (NCN) [grant 218/29/N/NZ9/00854]. KG was supported by the National Science Foundation USA award IOS-2142993 (CAREER). The funders had no role in study design, data collection and analysis, decision to publish, or preparation of the manuscript.

[Note: HTML markup is below. Please do not edit.]

Reviewers' comments:

Reviewer's Responses to Questions

**Comments to the Author**

1. Is the manuscript technically sound, and do the data support the conclusions?

The manuscript must describe a technically sound piece of scientific research with data that supports the conclusions. Experiments must have been conducted rigorously, with appropriate controls, replication, and sample sizes. The conclusions must be drawn appropriately based on the data presented.

Reviewer #1: Yes

Reviewer #2: Yes

2. Has the statistical analysis been performed appropriately and rigorously?

Reviewer #1: Yes

Reviewer #2: Yes

3. Have the authors made all data underlying the findings in their manuscript fully available?

The [PLOS Data policy](http://www.plosone.org/static/policies.action#sharing) requires authors to make all data underlying the findings described in their manuscript fully available without restriction, with rare exception (please refer to the Data Availability Statement in the manuscript PDF file). The data should be provided as part of the manuscript or its supporting information, or deposited to a public repository. For example, in addition to summary statistics, the data points behind means, medians and variance measures should be available. If there are restrictions on publicly sharing data—e.g. participant privacy or use of data from a third party—those must be specified.

Reviewer #1: Yes

Reviewer #2: Yes

4. Is the manuscript presented in an intelligible fashion and written in standard English?

PLOS ONE does not copyedit accepted manuscripts, so the language in submitted articles must be clear, correct, and unambiguous. Any typographical or grammatical errors should be corrected at revision, so please note any specific errors here.

Reviewer #1: Yes

Reviewer #2: Yes

5. Review Comments to the Author

Please use the space provided to explain your answers to the questions above. You may also include additional comments for the author, including concerns about dual publication, research ethics, or publication ethics. (Please upload your review as an attachment if it exceeds 20,000 characters)

Reviewer #1:

The manuscript entitled "Exploring Chilling Stress and Recovery Dynamics in C4 Perennial Grass of Miscanthus sinensis," submitted by Sobańska et al., presents a comprehensive study aimed at understanding the response of different Miscanthus sinensis genotypes to chilling stress and their recovery dynamics. This research is crucial for enhancing the agricultural viability and biomass production of Miscanthus in cooler climates, given its potential as a sustainable bioenergy source. The study meticulously evaluates 18 genotypes for their growth, chlorophyll fluorescence, and overall chilling tolerance, introducing a novel Total Chill Stress Response Index (SRI) that integrates various physiological and growth parameters. This index aids in identifying genotypes with enhanced chilling tolerance, thereby contributing significantly to the development of more resilient bioenergy crops.

We would like to sincerely thank Reviewer #1 for the valuable comments on our work and positive assessment of our work*.*

Clarification on Experimental Conditions:
Light and Chilling Stress Interaction: Please clarify under what light conditions (presence or absence of light) the plants were subjected to chilling stress. The impact of light on photosynthetic machinery, especially PSII under stress, is crucial and should be distinctly addressed to understand the pathways affected by these conditions.

A: The plants were grown under the light conditions described in the Materials and Methods section and in Fig 1A. Specifically “They were grown in a greenhouse under white fluorescent tubes, providing a 12-hour photoperiod of photosynthetically active radiation at 750 µmol m-2 s-1 light intensity at canopy level, with temperatures maintained at 25°C during the day and 19°C at night, and relative humidity at 60% ± 10%” (Fig. 1A) (Revision manuscript with track changes).

Ambiguities in Text:
Lines 95-107: These lines are ambiguous and need clearer expression. Please specify the details mentioned in this section to enhance understanding.

A: Thank you for this comment. We agree that the text lacked clarity. The section has been rewritten in the revision manuscript with track changes version (Line 111-131).

Lines 241-263: Similar to the above, this section lacks clarity in the explanation provided. It would benefit from a more straightforward presentation of the information.

A: Thank you for this comment. We agree that the text lacked clarity. The section has been rewritten in the revision manuscript with track changes version (Line 282- 312 , Revision manuscript with track changes).

Light Intensity Parameters:
Lines 135-153: The manuscript mentions a light intensity of 750 µmol m^-2 s^-1, which is significantly higher than the typical 300-500 µmol m^-2 s^-1 for C3 and C4 plants. Was this choice based on prior studies, and could this high intensity influence photoinhibition alongside chilling stress? Please provide a rationale or references to support this decision.

Chilling tolerance is most critical in spring and early fall when the combination of high light with chilling causes photoinhibition. Furthermore, the light conditions in that leaves develop are strictly related to photosynthetic capacity via adjustment of chlorophyll content and stoichiometry of antenna complexes to light conditions. To perform our experiment in the light conditions which field-grown plants might experience in spring and early fall we chose 750 µmol m^-2^ s^-1^.

The new text has been added to the Material and Methods section clarifying the choice of light conditions: “The experiment was performed under 750 µmol m^-2^ s^-1^ of light to mimic the light conditions which field-grown plants might experience in spring and early fall when chilling events occur.” (Line 163-165, Revision manuscript with track changes).

Technical Corrections:
Line 193 (PIABS Notation): Ensure that all scientific terms and abbreviations are correctly written throughout the manuscript to maintain professional and technical accuracy. For instance, PIABS should be uniformly formatted.

A: Thank you for this comment. In the revised version of the manuscript, we made sure that the scientific terms and abbreviations are correctly written and uniformly formatted throughout the manuscript. (Revision manuscript with track changes).

Methodology Description:
Lines 201-226: The use of a mixed linear model in the methods section is mentioned but not elaborated upon. Could you provide more details on its application and rationale?
Also, the parameters such as SRI and related ones need more details and should be presented more clearly.

A: Thank you for this comment. Methodology description has been extended by adding clarification, rationale and formulas. In response to this comment the new text has been added to the revision with track changes version of the manuscript in the lines 247-250, 261-263.
Data Visualization:
Results Section: A spider plot could be beneficial to visually contrast the most and least tolerant genotypes based on photosynthetic parameters. This would allow for an immediate, comparative visual representation of the data.

A: Thank you for this suggestion. The radar plots for relative photosynthetic parameters for treatment and recovery have been added to the revised version of the manuscript (S9 Fig)**.** The section has been rewritten to make clearer the reference to future work (Lines: 408, 412, 417, 421, 423, 433, 438,444, 447, 450, 456, 586, 613, 625) (Revision manuscript with track changes).

Streamlining the Results:
General Over-detailing: The results section is overly detailed, often repeating information that is also shown in figures. Aim to highlight key findings succinctly, focusing on significant results that advance the understanding of chilling stress responses.
Lines 373-382: This specific portion is too wordy. Simplify the text to focus on pivotal results and their implications, removing extraneous details.

A: Thank you for this comment. We trimmed and rewrote the text to concisely highlight key findings and focus on significant results. (Line 269-491, Revision manuscript with track changes).

Discussion Section Improvements:
Focus and Repetition: Avoid redundant presentation of data that was detailed in the results. Instead, focus on discussing the implications of these findings, the mechanisms involved, and how they relate to existing literature.
Lines 419-446 and 430-446: These sections repeat information and lack clear linkage to broader scientific concepts. They should be revised for clarity and relevance.
Lines 447-481: Ensure that all claims are supported by robust references and evidence. This is crucial for maintaining scientific credibility.

A: Thank you for this comment. We have trimmed and rewrote the text to focus on the implications of our findings, link them with broader scientific concepts and add references to evidence when applicable (Lines 496-749, Revision manuscript with track changes).

Conclusion and Terminology:
Lines 528-533 and 536-550: The conclusions drawn here appear speculative without sufficient empirical support. Clarify these assertions and ensure they are backed by the results presented.

A: Thank you for this comment. The text has been rewritten in the revision with track changes of the manuscript (Line 752 - 766).

Lines 559, 568-572: The mention of "molecular genetic techniques" and "high-throughput genome editing techniques" seems out of context as these were not part of the experimental approaches described. Either remove these terms or clarify if they refer to potential future work.

A: This section was intended to present new perspectives in the next research on chilling stress resulting from the selection of genotypes with varying tolerance to cold, including the application of high-throughput genome editing techniques. The section has been rewritten to make clearer the reference to future work (Line 757 - 762, Revision manuscript with track changes).

Technical and Editorial Corrections:
References and Formatting: The reference section requires thorough revision to ensure all citations are correctly formatted and relevant. Additionally, scientific names should be italicized, and abbreviations like CO2 should be consistently presented in the correct format.

A: We apologize for the mistakes in formatting the reference list. The list has been corrected in the revised version of the manuscript (Line: 788-954, Revision manuscript with track changes).

Deepening the Analysis:
Consider including a correlation matrix of the parameters to discuss the physiological responses more deeply, especially in the most sensitive and tolerant genotypes. This could provide insights into the interconnected pathways affected by chilling stress.

A: Thank you for this suggestion. We have added a correlation matrix (S1 Fig) to the revised version of the manuscript which extends the basic information about the degree of correlation that was already presented on biplot (Line: 313-318; 421-422; 455-460, Revision manuscript with track changes).

General Suggestions:

Reduce verbosity across the Results and Discussion sections and sharpen the focus on mechanistic insights and physiological implications.
The impact of chilling stress on photosynthetic parameters, particularly relating to PSII and electron transport chains, should be more explicitly connected to the observed physiological responses. Also, shouls consider the fact that during the chilling stress, the combination of reactions can cause the stress which in ETC the pool of NADP+/NADPH or the capacity if the ETC should be consider. Just mentioning the NPQ reactions without any proof is not a reliable way!

A: In this study, we mainly focused on developing a tool - the Total Chilling Stress Index (SRI) - that enables a comprehensive selection of genotypes that are more resistant to cold. The text of the Results and Discussion section has been substantially trimmed. We also re-worded the text to make a more explicit connection between measured parameters and plant physiological response (Lines 630-749, Revision manuscript with track changes).

Reviewer #2: This research has considered appropriate assumptions. Data analysis is done correctly. The results are new and noteworthy, and it has also provided a suitable discussion. Therefore, its publication is recommended.

Thank you for your recommendation.

6. PLOS authors have the option to publish the peer review history of their article ([what does this mean?](https://journals.plos.org/plosone/s/editorial-and-peer-review-process#loc-peer-review-history)). If published, this will include your full peer review and any attached files.

If you choose “no”, your identity will remain anonymous but your review may still be made public.

**Do you want your identity to be public for this peer review?** For information about this choice, including consent withdrawal, please see our [Privacy Policy](https://www.plos.org/privacy-policy).

Reviewer #1: No

Reviewer #2: No

[NOTE: If reviewer comments were submitted as an attachment file, they will be attached to this email and accessible via the submission site. Please log into your account, locate the manuscript record, and check for the action link "View Attachments". If this link does not appear, there are no attachment files.]

While revising your submission, please upload your figure files to the Preflight Analysis and Conversion Engine (PACE) digital diagnostic tool, <https://pacev2.apexcovantage.com/>. PACE helps ensure that figures meet PLOS requirements. To use PACE, you must first register as a user. Registration is free. Then, login and navigate to the UPLOAD tab, where you will find detailed instructions on how to use the tool. If you encounter any issues or have any questions when using PACE, please email PLOS at [figures@plos.org](mailto:figures@plos.org). Please note that Supporting Information files do not need this step.
